# Sowing Mixtures of Native Plant Species: Are There Any Differences between Hydroseeding and Regular Seeding?

**DOI:** 10.3390/plants10112507

**Published:** 2021-11-18

**Authors:** Vilma Gudyniene, Sigitas Juzenas, Vaclovas Stukonis, Egle Norkeviciene

**Affiliations:** 1 Lithuanian Research Centre for Agriculture and Forestry, LT-58344 Kedainiai, Lithuania; vilma.gudyniene@lammc.lt (V.G.); vaclovas.stukonis@lammc.lt (V.S.); 2 Institute of Biosciences, Life Sciences Center, Vilnius university, LT-10257 Vilnius, Lithuania; sigitas.juzenas@gf.vu.lt

**Keywords:** legumes, grasses, forbs, plant cover, species richness

## Abstract

Hydroseeding is a convenient, low-cost way to plant seeds. Traditionally, fast-growing commercial species that are cheap to obtain are preferred in hydroseeding, while native species have limited use. Nowadays, the use of native species is often desired in revegetation projects. However, there is a paucity of information about hydroseeding native species in Northern areas of Europe. Therefore, we aimed to determine whether hydroseeding has any effects on native plant cover formation, species richness and abundance, the development of plant morphological features, or aboveground biomass. A total of 40 native plant species in Lithuania were sowed using hydroseeding and regular seeding. The experimental plots were assessed for two years. The results show a relatively small and short positive effect of hydroseeding on plant cover formation. No significant differences were found in species richness between the sowing treatments. However, a comparison of species composition revealed significant differences between the sowing treatments that were more associated with species abundance than species diversity. Hydroseeding was favoured by legume species, such as *Onobrychis viciifolia*, *Ononis arvensis*, *Lotus corniculatus*, and *Trifolium medium*, while *Festuca rubra* favoured the regular seeding treatment. Overall, our findings emphasize that legume species that display more competitive growth traits should be included in the seed mixture in lower proportions when hydroseeding is applied.

## 1. Introduction

Hydroseeding, or hydraulic seeding, originated in the United States in the 1950s as a successful method that helps to prevent soil erosion by stabilizing and revegetating disturbed areas [1]. Recently, hydroseeding technology has become the most widely used instrument suitable for inaccessible sites and slopes, such as road and rail embankments [2]. Moreover, this technique is widely used for sowing large areas and semi-natural meadows [3] and restoring damaged ecosystems and degraded areas, such as mining wastes [4].

Traditionally, commercial seed mixtures of fast-growing species (mainly grasses and legumes) are used for hydroseeding [5]. In common practice, these mixtures include commercial seeds (mostly varieties of exotic genotypes) with a low percentage of autochthonous species [6]. Therefore, commercial seeds are becoming an important part of the seed bank, which can also have long-term consequences for natural ecosystems [7]. Several studies in Southern Europe have documented that hydroseeded commercial species act as a starter species and can constrain vegetation dynamics in the long term [8,9]. For this reason, species of non-native origin should be avoided in commercial seed mixtures when the target is to enhance biodiversity and ecosystem stability [9]. Moreover, a study in Mediterranean Europe showed that sowing introduced commercial species can cause hydroseeding to fail because of the unsuitability of the species to long periods of drought and/or intense rainfall [10]. These findings suggest that the application of non-native species could be limited in certain (arid and semi-arid) environments [11]. This is an important issue for future research, and further studies with more focus on autochthonous species were suggested [6]. Seeding native plant species is increasingly recommended due to long-term environmental goals. Firstly, native species are adapted to local abiotic and biotic conditions. Secondly, they preserve regional biodiversity and ensure the success and sustainability of the restoration of the original habitat [12]. The ecosystem functions, provision of wildlife habitats, and aesthetic benefits are also important considerations [13]. Even though there is now a global trend of using wild plants in revegetation, drawbacks such as relatively low commercial availability, high costs of the seeds of native species, and insufficient knowledge of their ecology limit their common use in the establishment of urban meadows or green areas.

The use of native species in revegetation is often desired when addressing ecological issues, but there is little information regarding their sowing methodology, for instance, the optimal seed density on a particular site [13]. A search of the literature revealed that most studies have focused on the evaluation of plant establishment when hydroseeding is used [9,11,14,15]. Several attempts have been made to show changes in floristic composition after hydroseeding [14,16]. However, much uncertainty still exists about the impact of hydroseeding slurry components on plant germination and early development [11,17]. Some of the issues emerging from previous studies on the hydroseeding of native species relate specifically to seedling mortality and lower plant densities [9,11,18,19]. This could be explained by the improved environmental conditions of hydroseeding (e.g., high soil fertility and water availability), which can have the opposite effect on wild species [20,21]. According to a few authors, a focus on key plant species is therefore required while testing hydroseeding effectiveness [18,22]. However, the success of hydroseeding is unknown and highly dependable on local conditions, which suggests there is a need for specific pilot studies prior to determining hydroseeding as appropriate [6]. Studies that take these variables into account will need to be undertaken. Furthermore, there are no data that can provide more information about the hydroseeding of native species in northern areas of Europe, and the effect of hydroseeding on wild species growing in hemi-boreal meadows remains ambiguous.

The aim of this study was to determine whether hydroseeding vs. regular seeding has any specific effects on grass cover formation and species richness and abundance, as well as the development of plants and aboveground biomass partitioning of native plant species of Lithuania. Moreover, we compared the temporal variations while assessing the species richness and composition in the experimental plots before mowing in summer and in the aftermath in autumn. Improved knowledge on the use of native species in hydroseeding is expected to fill some of the gaps in information on revegetation agronomy and ecology.

## 2. Results

### 2.1. Plant Cover and Species Richness

In the current study, significant differences (Mann–Whitney U test, *p* < 0.05) were found in plant cover among the plots sown using hydroseeding (HS) and regular seeding (RS). In the first year, the plant cover at 2 cm in height from the soil attained 94.0% (95% confidence interval (CI), 91.5–96.1) with RS and 97.4% (95% CI, 96.6–98.0) with HS. In the second year, the plant cover was denser in both sowing treatments with 97.5% (95% CI 96.2–98.6) with RS and 100.0% (95% CI 100.0–100.0) with HS, and the difference between the treatments remained significant (Mann–Whitney U test, *p* < 0.01). Further analysis showed that HS provided a more uniform plant cover (coefficient of variation (CV) = 1.29) compared to RS (CV = 4.19) in the first year, whereas the differences levelled off in the second year (HS CV = 0.0; RS CV = 2.12). However, the Fligner–Killeen test clearly indicated a significant difference in the variability (CV) of plant cover between the RS and HS plots in the second year as well (first year: *p* = 0.04; 2nd year: *p* < 0.01). Further analysis of the aftermath showed that the plant cover reached more than 99% in both sowing treatments, and no significant (*p* < 0.05) differences in the variability of the plant cover between the HS and RS plots were found. The plant covers of single species in the first year are presented in Table 1; Table 2 presents the second year. 

Throughout the two years of the study, a total of 47 species, including germinated weeds, were detected across all study plots. Table 1 and Table 2 show the overall number of germinated species, which amounted to 27 (67.5%) of the 40 total sown species (the full list of sown species is given in Table 3). It was impossible to evaluate the species richness in the first year because the identification of grasses to species level was not successful in the first growing season. However, all eight (100.0%) species of sown legumes and eight (34.7%) species of sown forbs were identified in the plots in the first year. The results, as shown in Table 1 and Table 2, indicate that all species of legumes overwintered and no differences in legume richness were observed in the second year. Six species (66.6%) of sown grasses were identified in the study plots in the second year, while *Avenula pubescens*, *Koeleria glauca*, and *Phleum phleoides* did not germinate and, therefore, were not detected. Comparing forbs species from the first and second years, a change in species composition was found. Even though the richness of forbs was very similar in both years and amounted to eight (34.7%) species in 2020 and nine (39.1%) in 2021, the shared species for both years were *Agrimonia eupatoria*, *Galium verum*, *Leucanthemum vulgare*, and *Plantago media*. The next four species, *Origanum vulgare*, *Salvia pratensis*, *Thymus pulegioides*, and *Vicia cracca*, germinated and were detected only in the first year, and the other four species, *Betonica officinalis*, *Campanula glomerata*, *Scabiosa ochroleuca*, and *Verbascum nigrum*, germinated and were identified only in the second year (Table 2). A total of 10 (43.0%) species of forbs were not germinated and not detected in the study years, including *Anthericum ramosum*, *Armeria maritima*, *Centaurea scabiosa*, *Clinopodium vulgare*, *Galium boreale*, *Gentiana cruciata*, *Knautia arvensis*, *Leontodon hispidus*, *Silene nutans*, and *Veronica longifolia*. The single most striking observation to emerge from the data comparison was that with the sowing of the core species (grasses and legumes) at seed densities over 200 times higher (7985.5 pure live seeds (PLS)/m^2^ and 436.9 PLS/m^2^, respectively) than the subordinate species (forbs 41.7 PLS/m^2^), the latter were not suppressed and germinated in the second year after sowing. 

**Figure 1:**
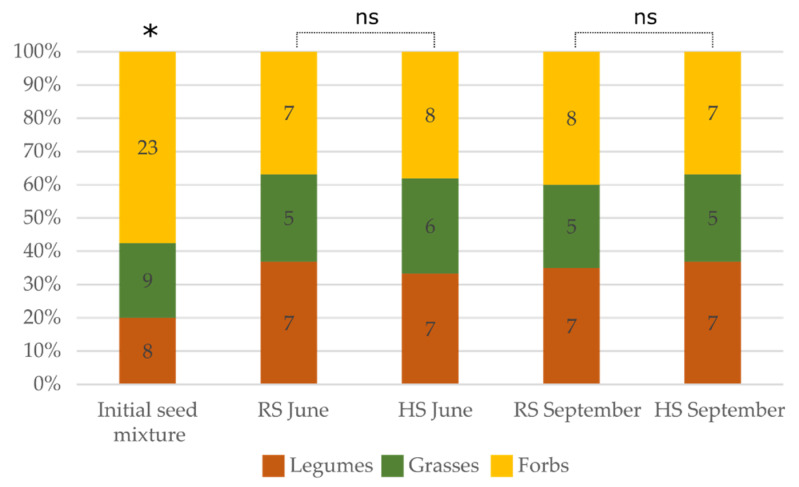
The species richness of initial seed mixture and in study plots (HS—hydroseeding, RS—regular seeding) before plant mowing (June) and in the aftermath (September) in the second year of growth. The numbers in the columns indicate the number of species. Initial seed mixture was compared to all treatments, and significant differences were detected in each variant. *—Statistically significant difference; ns— not statistically significant difference (diversity permutation test).

Figure 1 presents the species richness of the initial seed mixture vs. the sown experimental plots of HS and RS in June (before mowing) and September (aftermath) in the second year of growth. Diversity permutation testing indicated significant differences (*p* < 0.05) between the initial number of species in the mixture vs. germinated species in the HS and RS plots. The number of germinated species in the HS plots amounted to 47.5% in June and 52.5% in September of the initial number of sown species, while in the RS plots, species richness amounted to 52.5% and 50.0%, respectively (Figure 1). However, statistically significant differences in the species numbers between the HS and RS plots were not observed in this study in either season (June *p* = 0.37; September *p* = 0.25).

A total of 20 weed species were identified during the second experimental year. Significantly lower species numbers (Diversity permutation test, *p* = 0.03) were noted in the HS plots compared to the RS plots in June. Nevertheless, no significant differences in weed numbers were detected between the HS and RS plots in September (diversity permutation test, *p* = 0.31). Most of the weed species across all plots were perennials (65.0%), and the rest of them were annuals. Eight species were common in both treatments, including *Cerastium holosteum*, *Cirsium arvense, Lolium perenne, Medicago lupulina*, *Plantago major*, *Senecio vernalis*, *Taraxacum officinale*, and *Trifolium pratense*. Two species, *Plantago lanceolata* and *Rumex crispus*, were characteristic of only the HS plots, whereas, in the RS plots, most of the characteristic weed species were annuals (*Capsella bursa-pastoris*, *Erigeron canadensis*, *Lactuca serriola*, *Matricaria discoidea*, *Poa annua*, *Sonchus arvensis*, and *V. polita*), along with three perennials—*Astragalus glycyphyllos*, *Pilosella* spp., and *Trifolium repens*.

### 2.2. Species Composition

**Figure 2:**
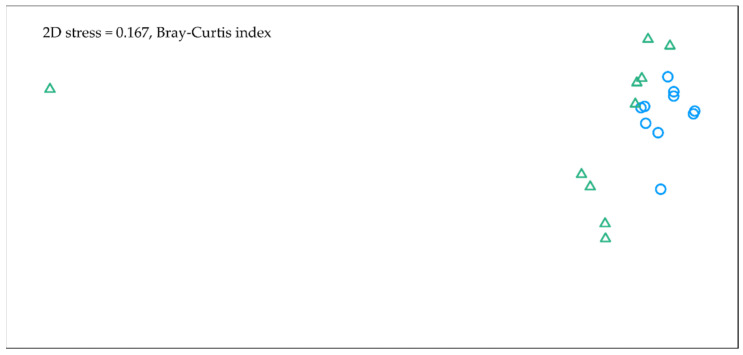
Non-metric multidimensional scaling (NMDS) of legumes and forbs species composition using dissimilarity measures of abundance data in hydroseeding and regular seeding in October in the first year of growth. Green triangles indicate—regular seeding, blue circles—hydroseeding.

The one-way PerMANOVA on the Bray–Curtis index performed on the first-year data revealed that the species compositions were significantly different between the HS and the RS treatments (F = 4.88, *p* < 0.05). However, the results reflect only the species composition of legumes and forbs because the grasses were excluded from multivariate analyses. The non-metric multidimensional scaling (NMDS) scatter plot presented in Figure 2 shows that more uniform species compositions were found in the HS compared to the RS plots. Further analysis indicated that statistically significant differences in the legume species compositions were more associated with species abundance (one-way PerMANOVA on the Bray–Curtis index, F = 4.74, *p* < 0.01) rather than species diversity (PerMANOVA on the Jaccard index, F = 1.81, *p* = 0.21). In the forbs group, both qualitative and quantitative differences in species compositions were significant (one-way PerMANOVA on the Jaccard index, F = 2.17, *p* = 0.02; Bray–Curtis index, F = 3.74, *p* = 0.02).

To determine which legume and forb species can be considered indicators of the sowing treatment (HS vs. RS) in the first year of growth, an indicator species analysis (IndVal) was performed. The test identified three species of legumes with significant indicator values in the HS plots—*Onobrychis viciifolia*, with an IndVal of 72.00%; *Ononis arvensis*, with an IndVal of 68.24; *Lotus corniculatus* with an IndVal of 68.09%. The forbs were not indicative of the performance of HS in the species composition, but *L. vulgare* (IndVal 51.43%), *G. verum* (IndVal 58.33%), and *Anthylis vulneraria* (IndVal 62.83%) negligibly indicated the performance of HS due to higher specificity (abundance); however, the IndVal values were not significant. The IndVal test was unable to identify the indicator species in the RS plots because none of the sown legumes and forbs had pronounced significance of their indicator values.

The results of the two-way PerMANOVA calculated with the data of the second year of growth revealed that the species compositions significantly differed between the sowing treatment (HS vs. RS) and the seasons (before mowing in June vs. in the aftermath in September), with a significant interaction of both factors (Table 3), which shows that differences in species compositions between the seasons were not uniform across the HS and RS plots. Nevertheless, taking into account the variations in species composition due to sowing treatment, greater variability was detected between the HS and RS plots in June rather than in the aftermath in September.

**Figure 3:**
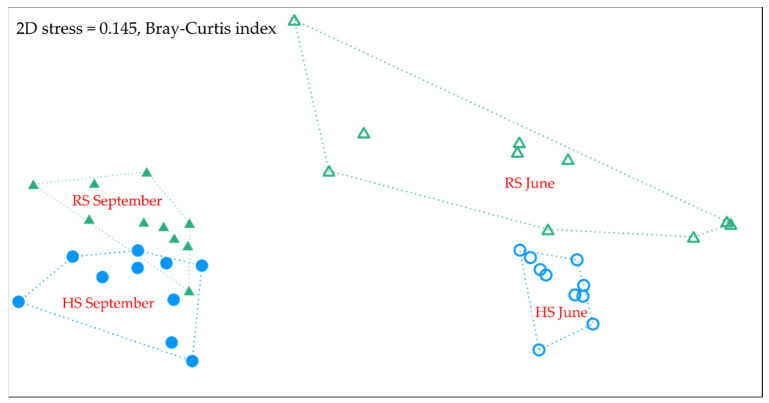
Non-metric multidimensional scaling (NMDS) of species composition using dissimilarity measures of abundance data in the hydroseeding (HS) and regular seeding (RS) plots in June (before mowing) and September (aftermath) in the second year of growth. Green triangles—regular seeding; blue circles—hydroseeding. Triangles and circles filled with colour indicate plots in September; unfilled—in June.

The NMDS scatter plot (Figure 3) clearly indicates that relatively similar and uniform species compositions were found in the plots of HS in June, whereas in the plots of RS, the species compositions were more variable in the same month, showing significant differences based on irregularities within the experimental plots. However, the NMDS scatter plot indicates that in the aftermath in September, differences between the species compositions of the HS and RS plots levelled off (Figure 3). Moreover, the species compositions became more similar within the same sowing treatment and between the sowing treatments. Pair-wise comparisons between the sowing treatments (HS vs. RS) were carried out separately in June (before mowing) and September (aftermath). The results show significant differences in the species compositions in both months (June: F = 7.551, *p* < 0.01; September: F = 4.362, *p* < 0.01).

The IndVal test was performed to determine which species can be considered an indicator of the performances of HS and RS in the second year. Of the 40 plant species studied, the IndVal test identified three species of legumes and one species of weed with a significant indicator value for HS in June—*L. corniculatus* (IndVal of 72.76%, *p* = 0.03), *O. arvensis* (IndVal of 63.91%, *p* = 0.03), *T. medium* (IndVal of 74.47%, *p* = 0.04), and *L. perenne* (IndVal of 80%, *p* = 0.03). In addition, the IndVal value of *Festuca rubra* was significant for RS in June (IndVal of 78.95%, *p* = 0.01). Notably, none of the species of sown grasses, legumes, or forbs had statistically significant fidelity or specificity between the HS and RS plots in the aftermath in September. Only the weed *L. perenne* had a statistically significant indicator value in the HS plots in the aftermath (IndVal of 86.36%, *p* < 0.01). 

Comparisons involving species composition in the same sowing treatment during different months displayed that *A. vulneraria* was a strong indicator of HS performance in June (IndVal of 97.51%, *p* < 0.01) due to its high specificity. However, it was not indicative of species composition in the aftermath. Regarding the species composition of HS plots in September, *A. cicer* (IndVal of 70.25%), *T. medium* (IndVal of 71.54%), and *F. rubra* (IndVal of 80.72%) had significantly higher specificity, thus making them statistically significant indicators in the aftermath. In turn, similar specificities were recorded for the RS plots between the seasons. Only two species were retained regarding the significance of their indicator values; *A. vulneraria* was a strong indicator in the RS plots in June (IndVal of 95.98%, *p* < 0.01), and *T. medium* had a specificity more pronounced in the RS plots in the aftermath (IndVal of 88.12%, *p* < 0.01).

### 2.3. Phenotypic Traits and Biomass

**Figure 4:**
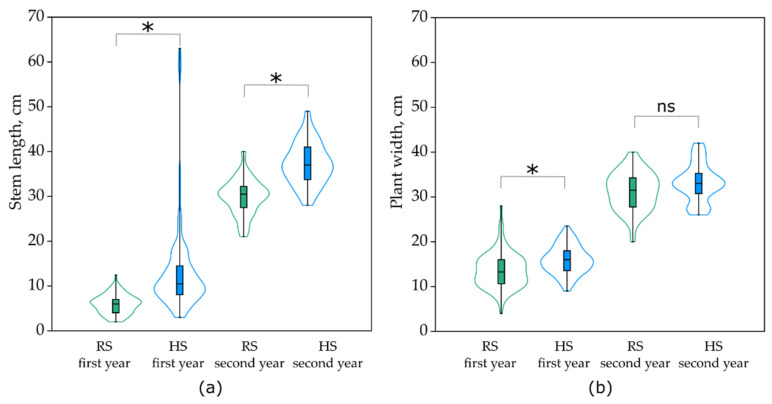
Stem length of *Onobrychis arvensis* (**a**) and plant width of *Anthyllis vulneraria* (**b**) in hydroseeded (HS) and regular seeded (RS) plots in the first (August 2020) and second (June 2021) growing years. Colour contours show kernel density plots from the minimum to the maximum value. Line in the box—median. *—Statistically significant difference; ns—not statistically significant difference (Mann–Whitney U test).

The stem lengths of *O. arvensis* and the plant widths of *A. vulneraria* were measured for two years to compare the plants’ vegetative growth between the HS and RS plots (Figure 4). The first-year results indicate the presence of statistically significant differences (Mann–Whitney U test, *p* < 0.05) in the stem length means of *O. arvensis* between the HS and RS plots. The stems of *O. arvensis* were twice as long in the HS (13.14 cm; 95% CI 11.25–15.11) than in the RS plots (6.0 cm; 95% CI 5.6–6.4). In the second year, the difference in stem length between the HS and RS plots was smaller (24.0% higher in the HS plots), though it remained statistically significant (Mann–Whitney U test, *p* < 0.05). 

Statistical nonparametric Mann–Whitney U testing revealed that the plant width of *A. vulneraria* was significantly (*p* < 0.05) greater in the HS plots (15.9 cm; 95% CI, 15.3–16.5) than in the RS plots (13.6 cm; 95% CI, 12.9–14.4) in the first year (Figure 4). However, the plant width did not differ (*p* > 0.05) between the HS and RS plots in the second year of study.

**Figure 5:**
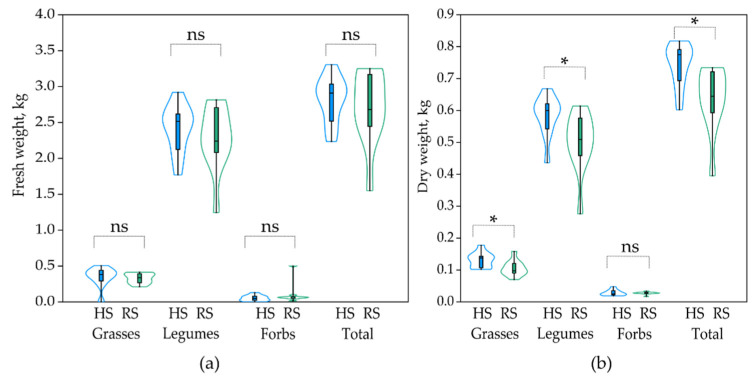
The biomass fresh (**a**) and dry (**b**) weight (kg/m^2^) partitioning between functional groups in hydroseeding (HS) and regular seeding (RS) plots in the second year of growth (July 2021). Colour contours show kernel density plots from the minimum to the maximum value. Line in the box—median. *—Statistically significant difference; ns—not statistically significant difference (*t*-test; Mann–Whitney U test).

In the current study, comparing the aboveground biomass partitioning among sown core (grasses and legumes) and subordinate (forbs) species groups under a single harvest system revealed that the legumes maintained their superiority over grasses and forbs and outyielded them (Figure 5a). However, a clear impact of the sowing treatment on the aboveground biomass fresh weight per plot could not be identified in this analysis, and none of the fresh weight differences between the HS and RS plots within a single group was statistically significant (*t*-test; *p* (perm.) > 0.05), for forbs biomass—Mann–Whitney U test, *p* > 0.05).

Contrary to the previous finding, in this study, we observed a significant difference in the biomass dry weight between the HS and RS plots (Figure 5b). The total biomass dry weight was 17.22% higher in the HS than in the RS plots (*t*-test; *p* (perm.) < 0.01). The dry weight of grasses in the HS plots was 23.81% higher compared to the RS (*t*-test; *p* (perm.) = 0.03); the same trend was noted for the legumes group—16.4% higher in the HS vs. the RS plots (*t*-test; *p* (perm.) = 0.03). However, the observed difference in the dry weight between the HS and RS plots was not significant for forbs (Mann–Whitney U test, *p* > 0.05).

## 3. Discussion

### 3.1. Plant Cover and Species Richness

Hydroseeding can achieve the establishment of vegetation cover in the short term by stabilizing the soil erosion, rebuilding soil, and improving the visual appearance of degraded sites [13]. Several studies performed in Mediterranean regions have shown that HS increased plant cover significantly [14,23], though the effect was not long-lasting. These are important aspects of ecosystem restoration. However, according to the study of García-Palacios et al. [9], HS was able to increase the total plant cover only by 10% and 16% while testing non-native plant species in Spain. However, other researchers showed that there were no significant differences in plant cover between HS and non-hydroseeded plots [6,19]. Our study results support the idea of previous findings, showing that HS did not have a strong impact on vegetation cover. This study was able to demonstrate only a 3.0% increase in the cover of native plant species in the HS plots in the first year. Nevertheless, the differences in plant cover levelled off in the second year of study. According to these data, we can highlight that HS can be considered to have a short-term positive value in providing native species plant cover in the early stages of vegetation development under hemi-boreal conditions.

Recommendations for the minimum vegetation cover for erosion control range from 20 to 80% depending on the site factors, such as slope, soil texture, and precipitation [13,24]. Under extremely high densities, plants can exhibit density-dependent mortality [13]. It is important to select the optimal seed rate without wasting the original input of seeds. According to Burton et al. [13], higher seed densities may not produce more plant cover because no difference was found in the cover formation in the first year between seed rates of 3.000 and 6.000 PLS/m^2^. In our study, hydroseeding native seeds at high rates of 8464.0 PLS/m^2^ (Table 4) provided more than 90% plant cover in all treatments in the first year. The cover in all treatments increased in the summer of the second year and levelled off in the aftermath in the autumn. Previous studies have demonstrated that plant cover increases until all available space is effectively occupied [13]. In our study, the high covers of the plots of all sowing treatments were determined by a high proportion of legumes in the mixture. However, some legume species, including *A. vulneraria*, have a lower persistence capacity [25], and mowing once per season weakens their domination in grassland. A further study with more focus on long-lasting plant cover differences between HS and RS is, therefore, suggested. Moreover, the site-specific optimal seed rate for the establishment of native herbaceous plant cover under hemi-boreal conditions needs to be identified. 

Another important finding was that HS resulted in a more uniform grass cover compared to RS. This is an important aspect for the visual appearance of plant cover and weed suppression. On the other hand, the presence of bare soil or uneven covering is an advantage for the creation of microsites without putting pressure on native biodiversity, thus avoiding landscape ecological homogenization. Microsites play an important role in plant establishment when seed availability is not limited [26,27]. Moreover, variability in the plant cover is favourable for differently sized and long-dormant seeds. In our study, significantly greater numbers of weed species were found with the RS treatments compared with HS. In general, the positive effect of HS on plant cover needs to be interpreted with caution if the introduction of local species and the maintenance of biodiversity are desired. 

As for species richness, this study did not detect any significant difference in species numbers between the HS and RS plots. These results match those observed in an earlier study in Mediterranean sites [6]. The most relevant finding was that the presence or absence of the species could be determined by the seed germinability, the species’ adaptive strategy, or the species’ ecological plasticity rather than the sowing treatment. According to these factors, we can tentatively divide species that are not germinated or detected into three groups—(1) species with a narrow ecological amplitude, confined to specific habitats, such as semi-natural dry grasslands (*A. pubescens*, *G. cruciata*, and *P. phleoides*), hydrophilous tall herb fringe grasslands (*V. longifolia*), xeric sand calcareous grasslands (*A. maritima*, *K. glauca*), or coniferous forests on or connected to glaciofluvial eskers (*A. ramosum*, *S. nutans*); (2) species with low germinability in the sowing mixture (*C. vulgare*, *G. boreale*, *L. hispidus*, and *V. longifolia*); (3) species that have adapted stress-tolerator strategies (*C. scabiosa*, *C. vulgare*, *K. arvensis*, *L. hispidus*, *O. vulgare*, *S. pratensis*, *T. pulegioides*, and *V. cracca*). Some studies have reported that plant species should be taken into account while testing the efficiency of hydroseeding [11]; however, an essential prerequisite is the use of a site-specific seed mixture [28]. Improved environmental conditions by hydroseeding mulches and organic amendments can have the opposite effect on wild species [19,20]. This is supported by our results—plant species confined to specific habitats did not germinate. 

Attention should also be paid to the specific seasonal preferences for germination [19]. Some species showed better germination in autumn than in spring [18]. This was shown in our study as well. Four species (*B. officinalis*, *C. glomerata*, *S. ochroleuca*, and *V. nigrum*) germinated and were detected only in the second year. Some species, such as *C. glomerata* and *V. nigrum*, had very low germinability (Table 4); nevertheless, they were not suppressed in the HS plots, where the plant cover and dominance of the core species were very high. Contrary to this finding, *O. vulgare*, *S. pratensis*, *T. pulegioides*, and *V. cracca* germinated in the first year but did not survive later. These species were weak competitors in the target growing conditions and were suppressed by other core legumes. The limited success of the germination of some forbs in the HS or RS plots might be partly explained by the significantly higher proportion of core species (legumes and grasses) in the mixture. Unbalanced proportions of legumes, grasses, and forbs, as well as higher proportions of species with morphological features (*A. vulneraria*, *O. viciifolia*, and *O. arvensis*), were apparently the major causes of such results. A reduction in the seed density is advisable because a reduced proportion of grass species in the mixture or the diversification of growth forms among grass species might contribute to a decreased dominance of generalist species in the first stages after seeding and allows for the establishment of other native species [19]. In general, our results support the findings of previous studies indicating that the species displaying more competitive growth traits should be included in lower proportions.

### 3.2. Species Composition

Previous reports have shown that HS can have small and short-term positive effects in providing species diversity for 2 years after its application [14]. The study of Ballesteros et al. [23] showed strong compositional and cover differences between hydroseeded and control plots. Matesanz et al. [6] noted that successfully established hydroseeded species did not decrease in abundance significantly over time; however, the species’ shifts in composition were extreme during the first years. Although the trends identified in our study are consistent with the data obtained in the above studies, they are difficult to compare with our findings due to the differences in species composition and hemi-boreal conditions. So far, the effects of HS on native species have not been closely examined in Lithuania. Therefore, this study revealed important insights into HS performance under hemi-boreal conditions. Other studies indicate that HS success is highly dependent on local conditions, suggesting the need for specific pilot studies prior to any large-scale initiative [6]. 

In the current study, the comparison of species compositions in the HS and RS plots revealed that significant differences were more associated with species abundance rather than species diversity. HS was favoured by legume species, such as *O. viciifolia*, *O. arvensis*, *L. corniculatus*, and *T. medium*. In terms of ecology, the higher abundance of legumes in grasslands has some positive features—(1) they are a source for many pollinators; (2) they fix atmospheric nitrogen, release high-quality organic matter in the soil, and facilitate soil nutrient circulation and water retention; (3) higher aboveground biomass suppresses weeds; (4) due to abundant flowering, they add an aesthetic value to the landscape. However, in our study, these legumes displayed more competitive growth traits and by acting as starter species, they outcompeted others. Matesanz et al. [6] found that hydroseeded *O. viciifolia* remained abundant for three years of study. If the species *O. viciifolia*, *O. arvensis*, *L. corniculatus*, and *T. medium* remained dominant over a longer period of time, this could cause a rapid decrease in plant evenness by competitive dominance and could suppress the introduction of local species. Consequently, in grasslands species, richness increases as a whole, while the contribution of sown species decreases [29]. More attention must be paid to species contributions in the sward because aggressive legumes can hinder the establishment or growth of other species of the mixture. These results emphasize the necessity for the careful calculation of the seed densities specifically for these legume species before HS is applied. The calculation of effective seed densities in HS mixtures should be based on the seed number rather than on the seed weight [19]. 

In terms of the species composition in the RS plots, *F. rubra* was more favoured by the RS treatment. Generally, the RS plots had more uneven grass cover with bare soil sites, leading to better growing conditions for *F. rubra* and non-target, early successional weed species. In terms of weeds, one unanticipated finding was that *L. perenne* was abundant in the HS plots. This weed was strongly indicative of HS performance and even increased in the aftermath in September of the second year. The occurrence of *L. perenne* was probably caused by the seeds left unintentionally in the HS tanks, thus explaining why it was more abundant than in RS plots. *Lolium* spp. has been reported as a highly competitive species [30]. Quality control of seed lots is necessary to avoid the unexpected substitution of a species, which may jeopardize efforts to apply a suitable mixture of native species [19]. 

Another important finding was that the species compositions in the experimental plots changed significantly from June to September when one mowing was performed. The seasonal variability in the species composition was greater than that caused by the sowing treatment. It is interesting to note that of all the species, only *A. vulneraria* was the most abundant in all plots in June. *A. vulneraria* is a biennial or perennial legume species and is known as a low-persistence herb in meadows or grasslands [31]. However, in the aftermath, a greater indicator value was shown by *A. cicer*, *T. medium*, and *F. rubra*. Monitoring the species composition and abundance is usually restricted to the first months after HS [5,6]. Further studies that take seasonal species diversity into account will need to be conducted in order to measure the HS success in hemi-boreal conditions. 

### 3.3. Phenotypic Traits and Biomass

Some studies have reported that HS mulches and organic amendments have a positive effect on improving soil properties, nutrient levels, and water conditions, thus increasing the plant cover and biomass in the short term [4,15,17]. Cellulose mulch sprayed together with fertilizers, organic amendments, and grass seeds will act as an absorbent mat that helps to maintain moisture around the seeds and accelerates their germination and growing process [32,33]. The results of this study indicate that HS had a positive effect on plant growth, though it was not long lasting. Higher values of plant morphological features in the HS plots were observed only in the first year. In the second year, the stem length of *O. arvensis* and the plant width of *A. vulneraria* were levelled. However, this experiment did not detect a significant difference in the plant aboveground biomass fresh weight between the HS and RS plots. Surprisingly, the dry weight per plot was significantly greater with the HS treatment. This result may be explained by the fact that there were higher proportions of legumes in the total biomass per plot. Additives included in the HS may have resulted in the growth of legume species and the accumulation of a higher dry matter weight. Fertilizer and other plant growth-promoters used in HS can result in the generalist species over others. García-Palacios et al. [9] suggested that fertilizer additions result in fast-growing species, which causes poor performance of the native species in HS plots in Mediterranean degraded areas. Our observations are consistent with the data obtained in previous studies; however, the trends need to be interpreted with caution due to the differences in species composition and hemi-boreal conditions. In general, it is expected that in the long term, the development of native plant species will not be markedly affected by HS application.

## 4. Materials and Methods

### 4.1. Study Area and Experimental Design

The study was carried out in 2020–2021 in the central lowland of Lithuania (55°23′49″ N; 23°51′40″ E), at the Lithuanian Research Centre for Agriculture and Forestry. The territory of Lithuania is unique from a geographical point of view because it is in an ecotone between two biomes. According to Lososová et al. [34], European boreal and nemoral biomes cross the territory of Lithuania. According to the environmental stratification of Europe, Lithuania is located in the Nemoral zone, with a cool temperate climate and a short growing season of 190–195 days [35]. The annual mean precipitation at the experimental site is 550–600 mm and the mean annual temperature is 6.0–6.5 °C. Climate records, as measured at the Dotnuva weather station, show that warm and rainy weather prevailed in June of the first experimental year. An agro-meteorological drought occurred in the second ten-day period of August. Due to the lack of precipitation, moisture reserves were not replenished and remained critical until October. The 2020–2021 winter season was cold with long-lasting snow cover. The spring was very late, cold, wet, and windy.

Based on the Lithuanian soil classification (LTDK-99), the soil at the study site is Endocalcari-Epihypogleyic Cambisol, with a moderately heavy loam texture predominates. According to the FAO soil classification the soil is Gleyic Cambisol [36] witha pH of 6.8, 18.4 g/kg^−1^ of organic carbon, and consists of 50.2% sand, 29.6% silt, and 20.2% clay in the 30 cm topsoil layer. In the autumn, the fields were ploughed, and in the spring before sowing, the weeds were controlled and the soil was harrowed, cultivated, and levelled with rakes. Pre-sowing fertilization was not used. Two experimental plots of 294 m^2^ were sown on 10 July using two methods—hydroseeding and regular seeding. Hydroseeding was performed by a commercial company using a FINN T120 HydroSeeder, and a homogeneous mixture of water, seeds, hydro-mulch, fertilizer, and chemical stabilizer was applied at high pressure (680 kPa) to the land surface without penetration into the deeper soil layers. A total of 7.7 L/m^2^ of hydroseeding slurry was added to each pot. The components of the slurry were wood/paper fibres mulch with a binder mixture (255.0 g/m^2^), green dye (6.0 mL/m^2^), surfactant (0.3 mL/m^2^), and fertilizer (NPK 15:15:15, 30 g/m^2^). Traditional seeding was carried out with a GANDY 36H13 seed spreader. After, the seeds were lightly embedded into the soil with rakes. The seeds with hydro-mulch vs. the seeds embedded into the soil surface were placed at the same depth (up to 1 cm deep). The seed rate of the mixture was 3.62 g/m^2^. No herbicides or additional fertilizers were applied after sowing.

The seeds of legumes and grasses for the experimental study were obtained from the Lithuanian Research Centre for Agriculture and Forestry. The seeds of forbs were collected in the natural habitats in Lithuania in 2018. The seed mixture was composed of 40 native plant species spontaneously growing in Lithuania. A major part of these species belongs to three vegetation classes—*Festuco-Brometea* (30.0% of all species), *Trifolio-Geranietea* (25.0% of all species), and *Molinio-Arrhenatheretea* (37.5% of all species). To retrieve information about the germinability of the seeds, 100 seeds of each of the species were germinated in the laboratory. The germinability was tested according to the methodology detailed by Aswathaiah et al. [37]. The results of the seed germinability test are presented in Table 4. A thousand seeds were counted with a seed counter Contador (Pfeuffer GmbH, Kitzingen, Germany), and the 1000-seed weight was measured with an electronic balance with a minimum accuracy of 0.01 g. A seed was considered a morphological unit of measure. The taxonomic nomenclature used is in accordance with the POWO [38] database. In Table 4 mentioned *Phleum pratense* L. was sown as *Phleum nodosum* L., but according to the POWO taxonomic nomenclature database, this is a synonym.

The plant species included in the sowing mixture were divided into two groups—core species (grasses and legumes) and subordinate species (forbs). All sown *Poaceae* taxa were considered grasses, and *Fabaceae* taxa were considered legumes. The sown plants of other taxa and weeds were considered forbs. The overall seed rate of the core grasses in the field was 1.99 g/m^2^ and accounted for up to 55.0% of the sown mixture’s weight; the core legume species—1.54 g/m^2^ and 42.5%, respectively—and subordinates—0.09 g/m^2^ and 2.5%, respectively. The total PLS was calculated by multiplying the percentage of pure seeds by the germination percentage, and then dividing the given value by 100. The results are based on 98% purity for each species. The seed rates of the tested plant species in the initial seed mixture are presented in Table 4.

### 4.2. Sampling

The assessments of field trials were carried out in the middle of October in the first year as well as at the beginning of June before mowing and in the aftermath at the beginning of September in the second year. The assessment of vegetation was carried out with the sampling plot method. A total of 10 sampling plots of 1 m^2^ (1 m × 1 m) were laid out randomly for each treatment. The cover of each plant species, as well as the cover of core grasses and the area of bare ground, were assessed with the percentage scale (from 0.1% to 100.0%) in each sampling plot. For more accurate measuring of the plant cover, a frequency grid divided into 100 squares was used. In the case of overlapping vegetation (such as a two-layer stand), only the visible portion of each layer was estimated. However, the identification of core grasses to species level was not successful in the first year, and therefore, the total coverage of all grasses was assessed. The species of core grasses were identified in the second year. The weed species in the sampling plots were identified in June and September in 2021 as well.

All plots were harvested on 5 July 2021, when 75% of the legumes and grasses had reached the flowering stage. The fresh weight of the biomass of all treatments was determined by weighing (kg) all live and dead plant tissue per 1 m^2^ plot. Lastly, the total biomass was divided into groups of legumes, grasses and forbs, and the fresh biomass of single group was weighted The dry matter yield was determined by drying all herbage samples at 105 °C to constant moisture.

Two species, *A. vulneraria* and *O. arvensis*, were chosen as model plants for evaluating the early development of the plants. The stem lengths of *O. arvensis* and widths of *A. vulneraria* plants were measured in each plot, with 100 randomly selected individuals. The measurements were done using a metal ruler with an accuracy of 1 mm. The first measurement was performed in August of the first year when the plants of the tested species were at the second principal growth stage (formation of side shoots and tillering). The second measurement was performed at the beginning of June in the second year when *O. arvensis* and *A. vulneraria* were at the booting growth stage. The principal growth stages were recorded following the method by Meier [39]. 

### 4.3. Statistical Analysis 

Student’s two-sample *t*-tests were performed. If the Shapiro–Wilk test confirmed normal distribution in the data and the non-parametric two-tailed (Wilcoxon) Mann–Whitney U test was used if the normal distribution was rejected. The 95% confidence intervals (CI) provided were obtained using the adjusted percentile method (BCa) with 99,999 bootstrapping replicates. The two-tailed Fligner–Killeen test was used to compare the variation in plant cover among sowing treatments. The diversity permutation test was used to compare the species numbers using 9999 random permutations.

Non-metric multidimensional scaling (NMDS) using the Bray–Curtis index was employed to compare species compositions in the sowing treatments [40]. A non-parametric one-way or two-way PerMANOVA test (based on the Jaccard or Bray–Curtis indexes, permutation *n* = 9999) was used to evaluate the difference in species composition between the experiment variants and seasons [41].

The indicator species analysis [42] method helped to assess which plant species were primarily responsible for an observed difference between the experiment variants. The species in the group can have an indicator value (IndVal) of 0 to 100%. In this work, the IndVal combined the species’ relative abundance (specificity) with its relative frequency of occurrence (fidelity) for a given variable (sowing treatment). The statistical significances of the indicator values were estimated by 9999 permutations of the sites across all groups.

All differences were considered significant when *p* < 0.05. The presented *p* values are Bonferroni corrected.

Statistical data analysis was performed using the computer program PAST version 4.07b [43].

## 5. Conclusions

Returning to the question posed at the beginning of this paper, it is now possible to state that hydroseeding (HS) has a relatively small and short-term positive effect on providing native plant species cover under hemi-boreal conditions. Another important finding was that HS resulted in more uniform grass cover compared to regular seeding (RS). This is an important aspect for the visual appearance of plant cover and weed suppression. The differences in plant cover levelled off in the second year of study. There were no significant differences found in species richness between the HS and RS treatments. The comparison of the species composition in the HS and RS plots revealed that significant differences were more associated with species abundance rather than species diversity. HS was favoured by legume species, such as *O. viciifolia*, *O. arvensis*, *L. corniculatus*, and *T. medium*, while *F. rubra* favoured the RS treatment. The limited success of some forbs’ germination with HS or RS might be partly explained by a significantly higher proportion of legumes in the mixture. Legume species that display more competitive growth traits should be included in the seed mixture in lower proportions. Our findings suggest that HS has a positive impact on plant growth compared to RS; however, the greater values of morphological features of the plants in the HS plots levelled off in the second year. The study did not detect a significant difference in the fresh weight of the plant aboveground biomass between the HS and RS plots, whereas the dry weight per plot was significantly greater with HS. These findings provide insights for future research of hydroseeding native plant species under hemi-boreal conditions. However, more research should be concentrated on investigating the seed rate, species performance over a longer period of time, and grassland maintenance when HS is used.

## Figures and Tables

**Table 1:** The frequency and plant cover of germinated plant species in the experimental plots (*n* = 10) in the first year.

Species		Hydroseeding		Regular Seeding	
		Frequency, %	Cover ^1^, %	Frequency, %	Cover ^1^, %
Legumes	*Anthyllis vulneraria*	100	22.9 (15–32)	100	13.6 (0.5–28)
	*Astragalus cicer*	100	6.4 (4–13)	100	5.5 (1–9)
	*Lotus corniculatus*	100	6.4 (4–10)	100	3.0 (1–5)
	*Onobrychis viciifolia*	100	0.9 (0.5–1)	60	0.6 (0.5–1)
	*Ononis arvensis*	100	5.8 (3–9)	100	2.7 (1–5)
	*Trifolium medium*	100	0.7 (0.5–1)	90	0.5
	*Trifolium rubens*	70	0.7 (0.5–2)	50	0.5
	*Vicia cracca*	0	0	10	0.5
Forbs	*Agrimonia eupatoria*	20	0.5	0	0
	*Galium verum*	100	0.7 (0.5–1)	100	0.5
	*Leucanthemum vulgare*	60	1.0 (0.5–2)	20	0.5
	*Origanum vulgare*	0	0	20	0.5
	*Plantago media*	10	0.5	0	0
	*Prunella vulgaris*	10	0.5	10	0.5
	*Salvia pratensis*	10	0.5	30	0.5
	*Thymus pulegioides*	10	0.5	0	0
Total grasses ^2^		100	50 (40–60)	100	44 (30–50)

^1^ The means of values recorded by visual observations for each species. In cases where the species were absent from the plots, they were not included in the calculation of the mean of the plant cover with the treatment. Minimal and maximal values are presented in brackets and given only when the variation in plant cover was measured. ^2^ Identification of grasses to species level was not successful in the sowing year; therefore, these values represent the frequency and the total cover of all grasses recorded by visual observation.

**Table 2:** The frequency and plant cover of germinated plant species in the experimental plots (*n* = 10) of hydroseeding (HS) and regular seeding (RS) in June before mowing of the plants and in the aftermath in September in the second year.

Species		HS, June		RS, June		HS, September		RS, September	
		Frequency, %	Cover ^1^, %	Frequency, %	Cover, %	Frequency, %	Cover, %	Frequency, %	Cover, %
Legumes	*Anthyllis vulneraria*	100	43.0 (30–55)	100	34.6 (10–60)	100	1.1 (0.5–2)	100	1.5 (0.5–2)
	*Astragalus cicer*	100	3.6 (2–6)	100	4.8 (2–7)	100	8.5 (5–15)	100	5.1 (3–9)
	*Lotus corniculatus*	100	21.1 (12–45)	100	7.9 (2–15)	100	14.5 (10–31)	100	13.9 (6–18)
	*Onobrychis viciifolia*	100	4.3 (1–7)	60	3.7 (1–10)	100	4.5 (2–8)	70	3.7 (2–7)
	*Ononis arvensis*	100	17.0 (12–26)	100	9.6 (4–17)	100	11.9 (5–18)	100	11.9 (6–20)
	*Trifolium medium*	100	3.5 (1–7)	100	1.2 (0.5–2)	100	8.8 (4–15)	100	8.9 (5–13)
	*Trifolium rubens*	100	1.8 (1–3)	100	1.4 (1–2)	60	1.4 (1–2)	50	1.1 (1–1.5)
Grasses	*Agrostis capillaris*	100	14.3 (10–20)	100	16.1 (10–20)	100	13.0 (5–20)	100	18.6 (12–22)
	*Festuca beckeri*	10	1.0	0	0	0	0	0	0
	*Festuca rubra*	100	1.6 (1–3)	100	6.0 (2–12)	100	6.7 (3–20)	100	9.8 (3–19)
	*Festuca trachyphylla*	90	0.6 (0.5–1)	100	2.6 (0.5–12)	90	1.4 (1–3)	90	2.0 (1–5)
	*Phleum pratense*	100	3.5 (2.5–5.5)	100	6.3 (3–10)	100	4.1 (2–6)	90	4.3 (1–8)
	*Trisetum flavescens*	100	11.7 (9–15)	100	6.3 (2–11)	100	12.5 (7–18)	100	13.4 (4–21)
Forbs	*Agrimonia eupatoria*	50	0.9 (0.5–1)	20	0.8 (0.5–1)	20	0.8 (0.5–1)	30	0.5
	*Betonica officinalis*	20	0.8 (0.5–1)	0	0	10	0.5	0	0
	*Campanula glomerata*	0	0	0	0	0	0	10	0.5
	*Filipendula vulgaris*	10	0.5	10	0.5	10	0.5	10	0.5
	*Galium verum*	80	0.6 (0.5–1)	100	1.1 (0.5–1.5)	70	1.4 (1–3)	100	1.8 (0.5–3)
	*Leucanthemum vulgare*	70	1.4 (1–3)	40	0.6 (0.5–1)	70	1.4 (1–2)	70	1.1 (0.5–2)
	*Plantago media*	20	0.5	10	0.5	10	0.5	10	0.5
	*Prunella vulgaris*	0	0	0	0	0	0	20	0.8 (0.5–1)
	*Scabiosa ochroleuca*	10	0.5	10	0.5	0	0	0	0
	*Verbascum nigrum*	40	1.0 (0.5–2)	50	0.5	40	0.9 (0.5–1)	30	0.8 (0.5–1)

^1^ The means of values recorded by visual observations for each species. In cases where the species were absent in the plots, they were not included in the calculation of the mean of the plant covers in the treatment. Minimal and maximal values are presented in brackets and given only when the variation in plant cover was measured.

**Table 3:** Two-way PerMANOVA test results of species composition of seeded plots with native plant species mixture, based on Bray–Curtis dissimilarity measures of abundance data, for seeding treatment (hydroseeding vs. regular seeding) and season (before mowing in June vs. aftermath in September). The *p*-values were obtained using 9999 permutations.

Source	SS	df	MS	F	p (perm)
Seeding	0.306812	1	0.30681	9.2479	0.0002
Season	1.16334	1	1.1633	35.065	0.0001
Seeding × Season	0.10024	1	0.10024	3.0214	0.0263
Residual	1.19434	36	0.033176		
Total	2.7647	39			

**Table 4:** The qualitative seed traits and seed rates of the investigated plant species.

Species	Germinability, %	1000 Seed Weight, g	Seed Rate, g/m²	Seed Density, n/m²
Legumes				
*Anthyllis vulneraria*	95	2.41	0.133	55.2
*Astragalus cicer*	85	3.62	0.480	132.6
*Lotus corniculatus*	90	1.23	0.116	94.3
*Onobrychis viciifolia*	90	21.39	0.163	7.6
*Ononis arvensis*	93	4.13	0.321	77.7
*Trifolium medium*	89	2.25	0.133	59.1
*Trifolium rubens*	95	2.73	0.182	66.7
*Vicia cracca*	43	2.76	0.012	1.6
*Total PLS ^1^/m^2^*	436.9			
*Grasses*				
*Agrostis capillaris*	98	0.08	0.340	4250.0
*Festuca beckeri*	4	2.56	0.010	3.9
*Festuca rubra*	62	0.55	0.255	463.6
*Festuca trachyphylla*	92	1.01	0.218	215.8
*Avenula pubescens*	95	0.96	0.510	531.3
*Koeleria glauca*	94	0.22	0.163	740.9
*Phleum pratense*	89	0.13	0.216	1661.5
*Phleum phleoides*	60	0.24	0.279	1162.5
*Trisetum flavescens*	41	0.45	0.085	188.9
*Total PLS/m^2^*	7985.5			
*Forbs*				
*Agrimonia eupatoria*	36	25.08	0.051	2.0
*Anthericum ramosum*	13	3.45	0.002	0.6
*Armeria maritima*	11	1.10	0.003	2.7
*Betonica officinalis*	69	1.20	0.003	2.5
*Campanula glomerata*	12	1.64	0.005	3.0
*Centaurea scabiosa*	46	0.97	0.012	12.4
*Clinopodium vulgare*	5	2.44	0.01	4.1
*Filipendula vulgaris*	66	0.76	0.005	6.6
*Galium boreale*	16	1.27	0.004	3.1
*Galium verum*	39	0.98	0.003	3.1
*Gentiana cruciata*	70	1.20	0.026	21.7
*Knautia arvensis*	50	0.88	0.002	2.3
*Leontodon hispidus*	8	4.00	0.003	0.8
*Leucanthemum vulgare*	42	1.68	0.005	3.0
*Origanum vulgare*	78	1.11	0.005	4.5
*Plantago media*	21	1.03	0.003	2.9
*Prunella vulgaris*	55	1.33	0.002	1.5
*Salvia pratensis*	43	1.42	0.005	3.5
*Scabiosa ochroleuca*	31	2.55	0.002	0.8
*Silene nutans*	36	1.62	0.002	1.2
*Thymus pulegioides*	13	0.82	0.007	8.5
*Verbascum nigrum*	9	1.12	0.010	8.9
*Veronica longifolia*	22	0.67	0.002	3.0
*Total PLS/m^2^*	41.7			

^1^ PLS—pure live seeds.

## Data Availability

The data presented in this study are available in the article.

## References

[1] Simcock R., Ross C., Smale M.C., Meurk C.D. (1995). Hydroseeding with New Zealand native species. Proceedings of the Workshop on Scientific Issues in Ecological Restoration.

[2] De Oña J., Ferrer A., Osorio F. (2011). Erosion and vegetation cover in road slopes hydroseeded with sewage sludge. Transp. Res. Part D Transp. Environ..

[3] Tamura N., Lulow M.E., Halsch C.A., Major M.R., Balazs K.R., Austin P., Kimball S. (2017). Effectiveness of seed sowing techniques for sloped restoration sites. Restor. Ecol..

[4] Albaladejo-Montoro J., Alvarez Rogel J., Querejeta J., Diaz E., Castillo V. (2000). Three hydro-seeding revegetation techniques for soil erosion control on anthropic steep slopes. Land Degrad. Dev..

[5] Bochet E., García-Fayos P. (2004). Factors controlling vegetation establishment and water erosion on motorway slopes in Valencia, Spain. Restor. Ecol..

[6] Matesanz S., Valladares F., Tena D., Costa-Tenorio M., Bote D. (2006). Early dynamics of plant communities on revegetated motorway slopes from southern Spain: Is hydroseeding always needed?. Restor. Ecol..

[7] González-Alday J., Marrs R.H., Martínez-Ruiz C. (2009). Soil seed bank formation during early revegetation after hydroseeding in reclaimed coal wastes. Ecol. Eng..

[8] Matesanz S., Valladares F. (2007). Improving revegetation of gypsum slopes is not a simple matter of adding native species: Insights from a multispecies experiment. Ecol. Eng..

[9] García-Palacios P., Soliveres S., Maestre F.T., Escudero A., Castillo-Monroy A.P., Valladares F. (2010). Dominant plant species modulate responses to hydroseeding, irrigation and fertilization during the restoration of semiarid motorway slopes. Ecol. Eng..

[10] Andrés P., Jorba M. (2000). Mitigation strategies in some motorway embankments (Catalonia, Spain). Restor. Ecol..

[11] Clemente A.S., Moedas A.R., Oliveira G., Martins-Loução M.A., Correia O. (2016). Effect of hydroseeding components on the germination of Mediterranean native plant species. J. Arid Environ..

[12] Nielsen A., Måren I., Rosef L., Kirkendall L., Malmstrøm M., de Boer H., Eldegard K., Hindar K., Hole L.R., Järnegren J. (2021). Assessment of Possible Adverse Consequences for Biodiversity When Planting Vascular Plants Outside Their Natural Range in Norway.

[13] Burton C.M., Burton P.J., Hebda R., Turner N.J. (2006). Determining the optimal sowing density for a mixture of native plants used to revegetate degraded ecosystems. Restor. Ecol..

[14] Martínez-Ruiz C., Fernandez-Santos B., Putwain P.D., Fernandez-Gomez M.J. (2007). Natural and man-induced revegetation on mining wastes: Changes in the floristic composition during early succession. Ecol. Eng..

[15] González-Alday J., Marrs R.H., Martínez-Ruiz C. (2008). The influence of aspect on the early growth dynamics of hydroseeded species in coal reclamation areas. Appl. Veg. Sci..

[16] Brofas G., Varelides C. (2000). Hydro-seeding and mulching for establishing vegetation on mining spoils in Greece. Land Degrad. Dev..

[17] Brofas G., Mantakas G., Tsagari K., Stefanakis M., Varelides C. (2007). Effectiveness of cellulose, straw and binding materials for mining spoils revegetation by hydro-seeding, in Central Greece. Ecol. Eng..

[18] Oliveira G., Nunes A., Clemente A., Correia O. (2011). Testing Germination of Species for Hydroseeding Degraded Mediterranean Areas. Restor. Ecol..

[19] Oliveira G., Clemente A., Nunes A., Correia O. (2013). Limitations to recruitment of native species in hydroseeding mixtures. Ecol. Eng..

[20] Jenkins A.M., Gordon D.R., Renda M.T. (2004). Native alternatives for non-native turfgrasses in Central Florida: Germination and responses to cultural treatments. Rest. Ecol..

[21] De la Riva E.G., Casado M.A., Jiménez M.D., Mola I., Costa-Tenorio M., Balaguer L. (2011). Rates of local colonization and extinction reveal different plant community assembly mechanisms on road verges in central Spain. J. Veg. Sci..

[22] Tormo J., Bochet E., García-Fayos P. (2006). Is seed availability enough to ensure colonization success? An experimental study in road embankments. Ecol. Eng..

[23] Ballesteros M., Cañadas E.M., Marrs R.H., Foronda A., Martín-Peinado F.J., Lorite J. (2017). Restoration of gypsicolous vegetation on quarry slopes: Guidance for hydroseeding under contrasting inclination and aspect. Land Degrad. Dev..

[24] Buss R.L., Brown C.S., Anderson J.H. (1997). Restoring native perennial grasses to rural roadsides in the Sacramento Valley of California: Establishment and evaluation. Restor. Ecol..

[25] Kazantseva E.S., Medvedev V.G., Onipchenko V.G. (2016). Estimation of the ontogeny stage durations for herbaceous plants, specifically *Anthyllis vulneraria* L. Ecol. Bull. North Cauc..

[26] Kiehl K., Kirmer A., Donath T.W., Rasran L., Hölzel N. (2010). Species introduction in restoration projects–evaluation of different techniques for the establishment of semi-natural grasslands in Central and Northwestern Europe. Basic Appl. Ecol..

[27] Vandvik V., Goldberg D.E. (2006). Sources of diversity in a grassland metacommunity: Quantifying the contribution of dispersal to species richness. Am. Nat..

[28] Krautzer B., Graiss W., Peratoner G., Partl C., Venerus S., Klug B. (2011). The influence of recultivation technique and seed mixture on erosion stability after restoration in mountain environment. Nat. Hazards.

[29] Muller S., Dutoit T.H., Alard D., Grevilliot F. (1998). Restoration and rehabilitation of species-rich grassland ecosystems in France: A review. Restor. Ecol..

[30] Hofmann M., Isselstein J. (2004). Effects of drought and competition by a ryegrass sward on the seedling growth of a range of grassland species. J. Agron. Crop Sci..

[31] Krikščiūnas J. (1942). Žemės ūkio Vadovas, Specialinė Žemdirbystė.

[32] Babcock D.L., McLaughlin R.A. (2013). Erosion control effectiveness of straw, hydromulch, and polyacrylamide in a rainfall simulator. J. Soil Water Conserv..

[33] Parsakhoo A., Jajouzadeh M., Motlagh A.R. (2018). Effect of hydroseeding on grass yield and water use efficiency on forest road artificial soil slopes. J. For. Sci..

[34] Lososová Z., Divíšek J., Chytrý M., Götzenberger L., Těšitel J., Mucina L. (2021). Macroevolutionary patterns in European vegetation. J. Veg. Sci..

[35] Metzger M.J., Shkaruba A.D., Jongman R., Bunce R. (2012). Descriptions of the European Environmental Zones and Strata.

[36] Food and Agriculture Organization of the United Nationals (2014). World Reference Base for Soil Resources: International Classification System for Naming Soils and Creating Legends for Soil Maps.

[37] Aswathaiah B., Gupta D.G., Ramegowda, Reddy M.V., Agrawal P.K. (1993). Germination testing. Handbook of Seed Testin.

[38] POWO (2021). Plants of the World Online. Facilitated by the Royal Botanic Gardens, Kew..

[39] Meier U. (1997). Growth Stages of Mono-and Dicotyledonous Plants.

[40] Taguchi Y.H., Oono Y. (2005). Relational patterns of gene expression via non-metric multidimensional scaling analysis. Bioinformatics.

[41] Anderson M.J. (2001). A new method for non-parametric multivariate analysis of variance. Austral Ecol..

[42] Dufrene M., Legendre P. (1997). Species assemblages and indicator species: The need for a flexible asymmetrical approach. Ecol. Monogr..

[43] Hammer Ø., Harper D.A.T., Ryan P.D. (2001). PAST: Paleontological statistics software package for education and data analysis. Palaeontol. Electron..

